# Can I Be a Better Dentist?

**Published:** 1916-04

**Authors:** Charles F. Ash

**Affiliations:** New York City


### CAN I BE A BETTER DENTIST?

BY CHARLES F. ASH, D.D.S., NEW YORK CITY.

What are the reasons which impel so many to attend our National and State meetings? A few attend solely for the recreation and good fellowship to be found, certain other few in the interest of dental politics, and a few others to see what new things the manufacturers have to offer; but it is reasonable to suppose that most of the men attending our conventions, go because they want to be better dentists. Otherwise why waste the time?

There are so many standpoints from which to view this subject that one hardly knows where to begin. Let me, in the first place, beg each man here to put the question seriously and honestly to himself. Can I be a better dentist?—with the emphasis on the word *can.* Now answer the question to yourself with Yes or No. *Can* I be a better dentist? I must assume that every man who has answered truly to himself has answered Yes. If then we *can* all be better dentists, the next question in natural sequence must be—Do you want to be a better dentist? Oh yes! Of course I want to be a better dentist—all right—now comes a crucial question. Why am I not a better dentist? Put this question squarely up to yourself—nobody else need know the answer—but at least be honest with yourself, tell yourself the real truth. This is not so easy, as we are all so prone to make excuses for ourselves that we rarely tell ourselves the exact truth; but now, be honest with yourself and what is the answer? If the answers were written out and collected, the tellers would report that the majority would be covered by two words—laziness and indifference. If either of these answers are true, then you have been your own jury and convicted yourself of dishonesty, for, if for any reason you are not giving your patients the best service of which you are capable, then you are not fair to your patients, and unfairness in this connection is synonymous with dishonesty. What I mean is this: Are your cavities properly prepared? Are they properly contoured? Are the fillings properly inserted? Are they properly finished at the margins and properly polished, or could you improve them a little in any one respect?

If you can improve them a little, then it's that little improvement for which I am pleading. But who puts in the other fillings? The ones we see every day, many of recent origin which we must condemn and remove and replace with better ones. Who left the little decay under those fillings? Who left a fringe of rough gold or amalgam under a gingival margin to start irritation and recession of the gums, retention of food pabulum and recurrence of decay? Who did that? Why it was the most elusive fellow in the world, commonly called "the other fellow."

Every conscientious operator, as his ideals and technique improve, sees some of his own work which he would like to do over again, that it might be made better, and for him I have no criticism. But I do criticise the man whose work is no better today than it was ten or five or even two years ago.

What is true of fillings and inlays is true also of every other branch of our work. Ill-fitting crowns and bands are made and inserted by the pound. Unscientific, dirt collecting and disease producing bridgework is still made and inserted by the ton. Crowns and fillings are still placed over root canals in dead teeth where no proper effort has been made to do thorough work.

Patients come into your offices every week with blind abscesses which are never even looked for, and fistulas which are never even found, or worse yet, they are discovered and not eliminated. More than half of all the devitalized lower first molars which I find, have no filling at all in the mesio-buccal canal and a large percentage of these show infections. A fine commentary on the intelligence of our profession. One might think that half the dentists didn't know there is such a thing as a mesio-buccal canal. A large percentage also of all the attempted root fillings I find are far short of the apex. Do you mean to tell me that the men who do these things don't know any better? I don't believe it.

As a result of the post graduate work in the sections of the First District Dental Society of the State of New York, most of the men have improved their equipment.

In the root canal section, nearly, if not quite every man has installed an X-ray outfit and many have also purchased apparatus for ionization treatment. No man who is filling root canals without the help of radiographs is doing himself or his patients justice. If you think that you are, just send the next half dozen patients for whom you fill root canals and have them radiographed (at your own expense if necessary), and see how many of them are filled to the apex and then just digest the thought that you are leaving a menace in every case where your filling comes short of the apex.

Is that the way you would want them in your own mouth? I doubt it. But in any case you have no right to leave them so in your patients' mouths. What shall you do? Why take them out, enlarge the canals, and fill them over again and then make another radiograph to make sure they are right.

If you have never done this I am sure you will find it a good investment. You will learn something about your own work which you never knew before, and should profit by it.

The time has come when the medical profession, through the work done by some of the more advanced men in the dental profession, have been aroused to the necessity for careful and scientific work in the mouths of their patients.

Every man here knows that we frequently work upon mouths in which there are diseased conditions, and yet let me ask what is done with the instruments after operating on such mouths? Only the merest pretense of sterilization is made. The instruments are probably washed with soap and water and perhaps passed through some further program of inefficient sterilization, such as dipping into alcohol for a few seconds or enclosing them in the average formalin sterilizer, or even putting them for a moment or two into the average steam sterilizer. The lack of efficiency in sterilizing in the average dental office is not due primarily to any desire to be negligent in this matter, but is due, rather, to a gross ignorance on the part of the profession as to what constitutes efficient sterilization. If you think that your present methods are efficient send some of your instruments to a bacteriologist after they have been passed through your usual form of sterilization and see what he finds—the results may be interesting. It is time that the profession learns something more about sterilization and improves its technique thereon.

To what extent are aseptic precautions followed when opening into a dental pulp? This is really a surgical operation and should receive the same surgical care that would be observed by a careful surgeon if he were cutting into a finger or some other portion of your anatomy, but this is not done. In the first place the field is rarely sterilized, and in many cases even the rubber dam is not used. Then in opening the cavities, stones and burs are used, which have been used in other mouths and not perfectly sterilized. I have even seen supposedly careful operators ask their assistant for a new bur and take the liberty of assuming because it was new it was sterile. The most eminent men, in both professions, are collaborating with the best bacteriologists in proving that many systemic diseases have their origin in infections in the mouth and around the roots of diseased teeth.

This work has just begun and will surely grow and develop. The family physician will ask you for a diagnosis of the pathological conditions in his patients' mouths. Are you prepared to give it? Are you prepared intelligently to discuss the case with him? Do you know how to make a culture? Do you even know how to take a smear for a culture?

The self-respecting dentist will prepare himself to meet this occasion, but what will happen to the other fellow? Are you "the other fellow?" If you are, for the love of your self-respect wake up! If you are the other fellow for the love of your fellowmen wake up. If you are the other fellow, for your own sake wake up!

For just as surely as you neglect to put yourself right in these matters just so surely will they come home to roost to your personal discomfiture, chagrin and loss.

Through the efforts of the best men in our profession, the medical men are being taught to demand those things. Are you going to wait until they are forced upon you? Ten thousand times No!

What are you going to do about it?

A few years ago I made a plea for comprehensive dentistry, and I have repeated it many times and I repeat it now.

The first thing to do is to bring your equipment up to a point of efficiency. This, in my opinion, should mean not only that our surroundings should be clean, but that we should have clean, sharp, absolutely sterile instruments; clean, sterile towels and napkins, an X-ray outfit and some method of minimizing pain, and last but not least—clean hands, for I have seen too many dentists operating with hands that I should hate to have put into my mouth.

Now what do I mean by comprehensive dentistry? I mean that when a patient presents, complaining of a pain in a R. U. first molar, you have no right to fill a possible cavity in that tooth and dismiss your patient and suppose that you have done your whole duty, even though you may have inserted a good filling. If you went to a physician and complained of a pain in your side, would he just give you something to stop the pain? No, if he is a careful man, he would first hear what you have to say and then proceed to make a comprehensive diagnosis. He would thereby increase the value of his services to you and also increase your respect for him.

A comprehensive examination of the mouth should include a careful search for cavities, for faulty fillings, crowns or bridges, for non-vital teeth, for faulty root canal fillings, for fistulas, for blind abscesses, for impacted teeth, for pyorrhea and faulty occlusion.

Where more or less extensive restorations are to be made, full upper and lower impressions should also be taken and models made. These models should be carefully studied in conjunction with radiographs of any diseased areas and a reasonably definite plan of campaign should be mapped out before doing any work in the patient's mouth. The general outline of this plan should be submitted to your patient, and he should be given to understand that you expect to take charge of his mouth and do for him whatever is necessary, and in nine cases out of ten, you will find that the patient is so interested and impressed by your thoroughness in diagnosis that he will be willing to submit himself to whatever you may prescribe as necessary. If you are not following these or similar steps in diagnosis, then you are not working in this regard to the limit of your present ability. You can be a better dentist right away by adopting this technique, also I predict that the results will be so satisfactory, both to you and your patients, that you will never again lapse into the old methods of slip-shod diagnosis.

The preparation of cavities or inlays is another step wherein I feel satisfied that a large percentage of the men can improve their work.

I wonder how many dentists, after preparing a cavity in a manner which seems to them satisfactory, take the trouble to look at the cavity through a magnifying glass? There is a glass made for the use of jewelers which is called a loupe and which I would recommend everybody to buy. It costs four or five dollars and the use of it will help any conscientious man to improve his technique.

If after preparing a cavity by your usual technique, you will look at it with a strong magnifying glass you will probably find that the margins of the cavity are not so smooth as you had supposed they were. You will also find possibly that there is some slight undercut and a correction of either or both of these conditions will undoubtedly improve your results. Such a glass will be found advantageous too when trimming the dies and also when trimming the inlays in the dies when following the indirect method. The carving and contouring of amalgam fillings and also the polishing of amalgam fillings, is a point where I know a large percentage of men fail to reach the limit of their present ability.

Then, too, the average gold shell crown comes far from properly fitting the tooth on which it is placed. This is due largely to the fact that the operator does not take sufficient time properly to prepare, cut down and trim the tooth for which the crown is intended, and if this first step is not properly done, then it is a physical impossibility to get even a fair fit. If this is the kind of crown you are making, then you are doing so because you are not working up to the limit of your present ability. It is not that the operator does not know better, but that he comes short of doing as good a piece of work as he is capable of doing.

If it is true that you are working up to the best of your present ability, then you can be a better dentist by increasing the limit of your ability, but this can not be done without some sacrifice of time, any more than any other good thing can be accomplished without proper effort. Post Graduate work is just as essential to the average practitioner, if he would grow, as are moisture and sunshine to seeds. I believe that it is desirable that every good dentist should take one or more dental magazines and read them and adopt such suggestions for new methods as may seem to be an improvement over his present methods. Post graduate classes for instruction have been formed in many of the larger cities, most of these under the auspices of the local society. Last year at our First District Society of New York, there were 125 men enrolled for post-graduate work with an average attendance of ninty-five per cent. all through the winter. Our classes included Orthodontia work, Prosthetic work, Crown, Bridge and Inlay work, Root Canal work, Oral Surgery, and next winter we intend to add a class in Bacteriology.

When I tell you that each section met twice a month and that many of the men attended two or three sections, and one or two attended all five sections, you will understand something of the earnestness of these men to increase their knowledge and improve the quality of their work.

I know that it is not always possible to run a series of classes in a comparatively small community, but in many cases, a man who is anxious to improve in one particular line, can readily find a sufficient number of others who will join him, so that they can start a class of their own, and with some expenditure of time and a small amount of money, could secure instructors. One thing, at least they could do, they could put their pride in their pockets and go to the man in their immediate neighborhood, whom they know excels them in this particular subject; frankly acknowledge their deficiency and ask him to take charge of a small class and teach them what he knows of that technique. This will accomplish a triple purpose. It will help you to improve your own work; it will stimulate to better efforts the man who is teaching you and will bring about a harmonious and get-together feeling in the neighborhood, and increase the feeling of good fellowship and mutual respect. Everybody respects a man who is honestly striving to learn, even though his present accomplishments may be below par.

In order to do his best work one must have enthusiasm, inspiration and imagination. The getting together of a few men united in one earnest purpose will foster the enthusiasm and inspiration, and one's own efforts to do better work will stimulate the imagination, for if a man would do some one thing with a better result than he has ever attained before, he must have in mind a mental picture of that which he is striving to achieve, and the higher the vision soars, the greater will be the achievement, and the higher one climbs the broader will be the vision.—*Journal Allied Dental Societies.*

